# GWAS and WGCNA Analysis Uncover Candidate Genes Associated with Oil Content in Soybean

**DOI:** 10.3390/plants13101351

**Published:** 2024-05-14

**Authors:** Xunchao Zhao, Yan Zhang, Jie Wang, Xue Zhao, Yongguang Li, Weili Teng, Yingpeng Han, Yuhang Zhan

**Affiliations:** Key Laboratory of Soybean Biology in Chinese Ministry of Education (Key Laboratory of Soybean Biology and Breeding/Genetics of Chinese Agriculture Ministry), Northeast Agricultural University, Harbin 150030, China; zhaoxunchao2017@163.com (X.Z.); zhangyan010122@163.com (Y.Z.); w15536351237@163.com (J.W.); xuezhao@neau.edu.cn (X.Z.); yongguangli@neau.edu.cn (Y.L.); twlneau@163.com (W.T.)

**Keywords:** soybean, genome-wide association study (GWAS), WGCNA, oil content

## Abstract

Soybean vegetable oil is an important source of the human diet. However, the analysis of the genetic mechanism leading to changes in soybean oil content is still incomplete. In this study, a total of 227 soybean materials were applied and analyzed by a genome-wide association study (GWAS). There are 44 quantitative trait nucleotides (QTNs) that were identified as associated with oil content. A total of six, four, and 34 significant QTN loci were identified in Xiangyang, Hulan, and Acheng, respectively. Of those, 26 QTNs overlapped with or were near the known oil content quantitative trait locus (QTL), and 18 new QTNs related to oil content were identified. A total of 594 genes were located near the peak single nucleotide polymorphism (SNP) from three tested environments. These candidate genes exhibited significant enrichment in tropane, piperidine, and pyridine alkaloid biosynthesiss (ko00960), ABC transporters (ko02010), photosynthesis-antenna proteins (ko00196), and betalain biosynthesis (ko00965). Combined with the GWAS and weighted gene co-expression network analysis (WGCNA), four candidate genes (*Glyma.18G300100*, *Glyma.11G221100*, *Glyma.13G343300*, and *Glyma.02G166100*) that may regulate oil content were identified. In addition, *Glyma.18G300100* was divided into two main haplotypes in the studied accessions. The oil content of haplotype 1 is significantly lower than that of haplotype 2. Our research findings provide a theoretical basis for improving the regulatory mechanism of soybean oil content.

## 1. Introduction

Soybean oil, as a main source of the human diet, is closely related to our daily lives [1]. Plant oil, also referred to as plant fat, primarily originates from the seeds of plants. The composition of vegetable oil primarily consists of five fatty acids, which collectively account for 98.4% of the total oil content, including palmitic acid (16:0), stearic acid (18:0), oleic acid (18:1), linoleic acid (18:2), and linolenic acid (18:3) [2,3]. Soybean oil is a prominent cooking oil and plays a pivotal role in disease prevention, so its synthesis mechanism has always been a research hotspot [4,5]. Therefore, revealing the characteristics associated with oil synthesis in plant seeds can help contribute to enhancing oil yield and quality.

In plants, the accumulation of seed oil primarily occurs in the form of triacylglycerol (TAG) [6]. The DGAT gene predominantly governs the process involved in triacylglycerol (TAG) synthesis. Research has found that overexpression of the DGAT gene significantly increases the accumulation of plant seed oil content [7]. In soybeans, *GmOLEO1* enhances oil accumulation by affecting the synthesis of triacylglycerol (TAG) [8]. The *GmSWEET39* gene exerts a positive influence on increasing the total oil content of soybeans and *Arabidopsis* [3]. Research has shown that the mitochondrial gene orf188 exerts an influence on the oil content of rapeseed [9]. Previous studies have demonstrated that WRI1, as a pivotal transcription factor regulating oil metabolism, can regulate key genes in glycolysis and fatty acid synthesis pathways, thereby exerting further influence on oil synthesis [10,11,12]. In addition, transcription factors such as LEC1, LEC2, MYB, ABI3, and bZIP play an important role in regulating seed oil accumulation [13,14,15,16]. With the development of high-throughput sequencing technology and genome-wide association analysis, it has become widely employed for the identification of QTLs/genes associated with various agronomic traits, for example, maize, soybeans, rice, and rapeseed. Currently, the identification of over 300 QTLs associated with seed oil content has been accomplished. A GWAS was employed to analyze the grain oil of 533 (305 indica subpopulation and 178 japonica subpopulation) rice accessions, where a total of 94 QTLs were identified to be associated with oil content, and the qPAL6 locus was detected for C16:0 composition [17]. A total of 3,290,923 SNPs were identified with 320 soybean accessions, 29 QTLs were identified to be significantly associated with oil content, while 24 loci are likely to be new [18]. Previous studies conducted GWAS analysis on 278 soybean materials using two models, and three significant QTLs were identified. Among them, the significant SNP (ss715637321 on chr20: 32835139) overlapped with the known large-effect oil QTL loci [19]. Duan et al. conducted a GWAS on over 1800 soybean materials, and the identification of a significant QTL locus on chromosome 5 controlling seed thickness was identified. Further investigation revealed that the allelic variation of the candidate gene GmST05 is the main factor affecting seed size in the soybean germplasm [20]. Qi et al. conducted GWAS analysis on the fatty acid content of 547 soybean materials, identified a significant SNP site on chromosome 9, and discovered a SEIPIN homologous gene that plays an important role in regulating fatty acid synthesis [21]. Li et al. conducted QTL mapping of oil content in the recombinant inbred line (RIL) population, and 5 QTLs related to oil content were identified. A total of 20 candidate genes were screened [22].

Soybean oil content is a quantitative trait that is governed by multiple genes. The GWAS based on natural populations has more recombination events compared to biparental populations, thereby leading to enhanced accuracy in phenotype association [23,24]. The GWAS has been employed to identify genomic regions associated with plant traits, including oil and protein content, yield, quality, and biotic and abiotic stress [25,26,27]. The weighted gene co-expression network analysis (WGCNA) enables the extraction of relevant genes from phenotype data and is extensively employed for investigating intricate relationships among different types of genes [28,29]. The WGCNA can be employed to further identify and prioritize potential candidate genes.

To elucidate the underlying mechanism of soybean oil biosynthesis, soybean oil content was analyzed through the GWAS using 227 soybean resources. Then, transcriptome data from 30 soybean seeds with high and low oil content were applied for WGCNA analysis. This study utilized the strategy of GWAS and WGCNA joint analysis to identify the putative regulatory genes governing oil content.

## 2. Results

### 2.1. Phenotypic Variation of Oil Content

**Figure 1:**
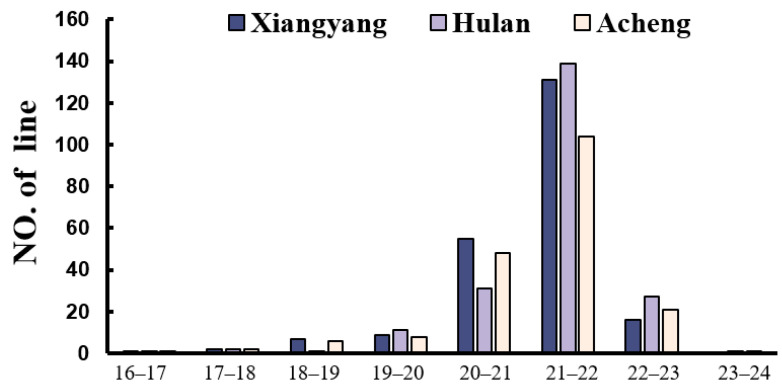
Frequency distribution of oil content in the three environments.

A total of 227 soybean resources were studied and the distribution is presented in Table 1. The oil content of the test population at three locations was 16.9–22.6% (Xiangyang), 17–23.8% (Hulan), and 17–24.6% (Acheng), respectively. The phenotypic variations of oil content were 4.18%, 3.62%, and 4.68% across three different environments (Xiangyang, Hulan, and Acheng), respectively (Figure 1, Table 1). The above results indicate that there are significant differences in oil content among the tested populations, as well as the abundant genetic diversity of germplasm resources, providing favorable conditions for the screening of specific germplasms.

### 2.2. Population Structure and GWAS Analysis

In this study, specific-locus amplified fragment sequencing (SLAF-seq) was applied to analyze 227 soybean germplasm resources. A total of 23,150 high-quality SNPs were selected (MAF > 0.05, missing data < 10%). The obtained SNPs are evenly distributed on 20 chromosomes of soybean (Figure S1A). By analyzing the principal components and phylogenetic relationships of SNPs, the changes in principal component analysis revealed an inflection point at PC3 (Figure S1B). These results showed that the first three phylogenetic relationships dominated the population structure on the association mapping (Figure S1C). Based on the pairwise relative kinship coefficients of association panel analysis, the 227 germplasm resources have low levels of genetic correlation (Figure S1D). The decay distance of LD is approximately 200 kb (Figure S2).

### 2.3. GWAS Identifies Significant SNPs Associated with Oil Content

**Figure 2:**
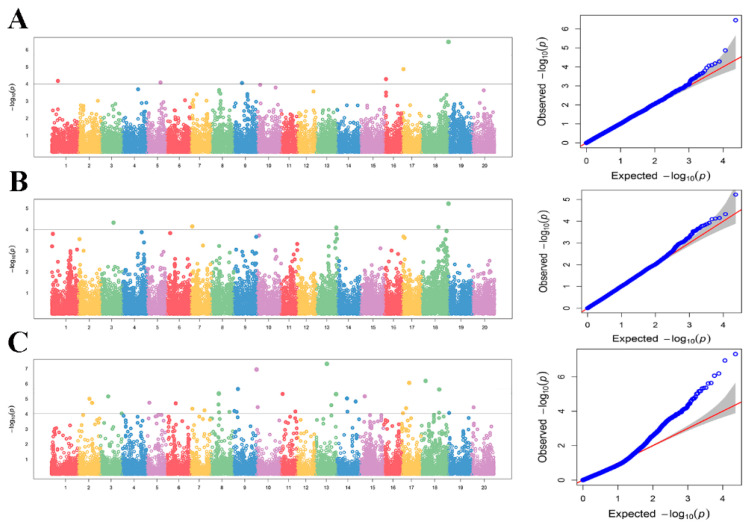
Manhattan plot and QQ plot of association mapping of oil content in soybean. (**A**) Xiangyang in 2019. (**B**) Hulan in 2019. (**C**) Acheng in 2019. The black line on each subgraph indicates the log10 (*p* value) significance threshold.

The genome-wide association study (GWAS) was analyzed using the compressed mixed linear model (CMLM) method. A total of 44 QTNs were found associated with oil content. The loci of significant QTNs are predominantly concentrated on chromosomes 1, 2, 3, 5, 6, 7, 8, 9, 10, 11, 13, 14, 15, 16, 17, 18, 19, and 20 (Figure 2). There are six significant QTN loci (rs334 on Chr01, rs5244 on Chr05, rs9475 on Chr09, rs16850 on Chr16, rs17991 on Chr17, rs20739 on Chr18) that are associated with oil content in Xiangyang (above the significance threshold −log10 (*p*) = 4). Four significant QTN loci (rs2905 on Chr03, rs6995 on Chr07, rs13933 on Chr13, rs20739 on Chr18) are associated with oil content in Hulan. However, there are the most significant SNP loci in Acheng, including 34 significant QTN loci. Meanwhile, one QTN locus (rs20739 on Chr18) was identified in two environments (Table 2).

### 2.4. Gene Enrichment Analysis of Candidate Genes

Gene enrichment analysis was performed to determine candidate genes for the regulating of oil content. The 200 kb genomic regions (100 kb on both sides) of each significant SNP locus in the GWAS results are identified as candidate genes. A total of 594 genes were located near the peak SNPs from three tested environments (Table S1). To further understand the potential function of candidate genes, the candidate genes were subjected to KEGG pathway analysis. These candidate genes exhibited significant enrichment in tropane, piperidine, and pyridine alkaloid biosynthesiss (ko00960), ABC transporters (ko02010), photosynthesis-antenna proteins (ko00196), and betalain biosynthesis (ko00965) (Figure S3). Among these identified candidate genes was *Glyma.18G299300*, an alpha/beta-Hydrolases superfamily protein located near rs20739 of Chr.18. This gene is an enzyme that promotes the hydrolysis of ester bonds between fatty acids and glycerol [43]. *Glyma.13G129900*, a GDSL-like Lipase/Acylhydrolase superfamily protein (located near rs13422 of Chr.13), plays a pivotal function of regulating seed oil content [44]. *Glyma.03G260300* (located near rs3413 of Chr.3), a 3-ketoacyl-CoA synthase 1 protein, has been proven to improve the synthesis of long-chain fatty acids in seeds [45]. *Glyma.18G299600* (located near rs20739 of Chr.18), a Phosphoenolpyruvate carboxylase family protein, has the function of negatively regulating oil content [46].

### 2.5. Identification of Key Modules Possessing Candidate Genes via WGCNA

**Figure 3:**
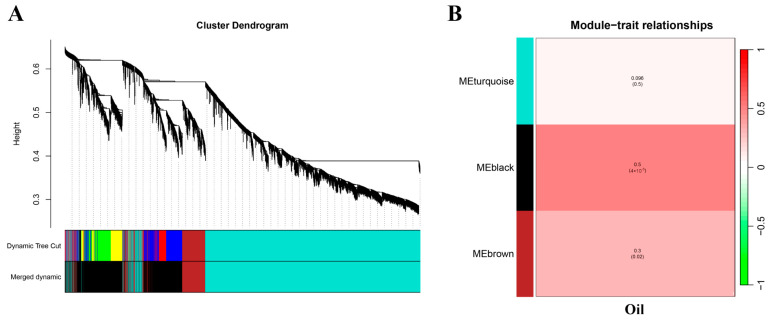
Weighted gene co-expression network analysis. (**A**) Clustering dendrogram of genes and construction of modules. (**B**) Phenotype and module correlation analysis heat map.

In order to further identify novel genes involved in the regulation of oil synthesis, transcriptome data from 30 soybean seeds with high and low oil content were applied for WGCNA analysis. Three modules were obtained in this study, represented by different colors in Figure 3A. Analysis of the relationship between modules–traits showed a relatively high correlation between one module and oil, including the MEblack module (r = 0.5, *p* = 4 × 10^−5^), where there are 863 genes strongly associated with oil content (Figure 3B).

**Figure 4:**
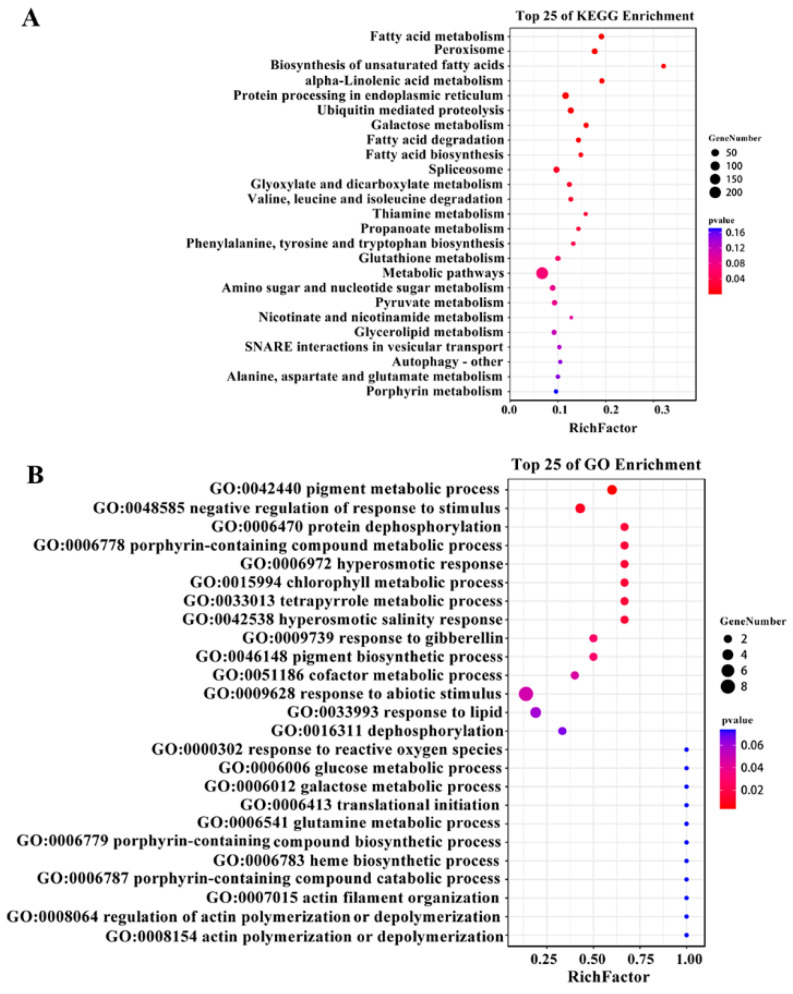
KEGG enrichment and GO annotations of the MEblack module. (**A**) KEGG enrichment of the MEblack module. (**B**) GO annotations of the MEblack module.

To understand the biological significance of co-expression networks, the genes in the MEblack module were subjected to Gene Ontology (GO) annotations and Kyoto Encyclopedia of Genes and Genomes (KEGG) enrichment analysis. The KEGG enrichment analysis revealed a significant enrichment of these genes in the metabolic pathways associated with fatty acid metabolism, fatty acid biosynthesis, fatty acid degradation, biosynthesis of unsaturated fatty acid, glycerolipid metabolism, pyruvate metabolism and alpha-linolenic acid metabolism (Figure 4A). Next, the present study performed GO annotations analysis on all genes within the MEblack module. As shown in Figure 4B, the most significant annotations terms were identified in the biological processes category, and the top five significantly enriched terms were found to be related to the pigment metabolic process (GO:0042440), a negative regulation of response to stimulus (GO:0048585), protein dephosphorylation (GO:0006470), porphyrin-containing compound metabolic process (GO:0006778), and hyperosmotic response (GO:0006972) (Figure 4B).

**Figure 5:**
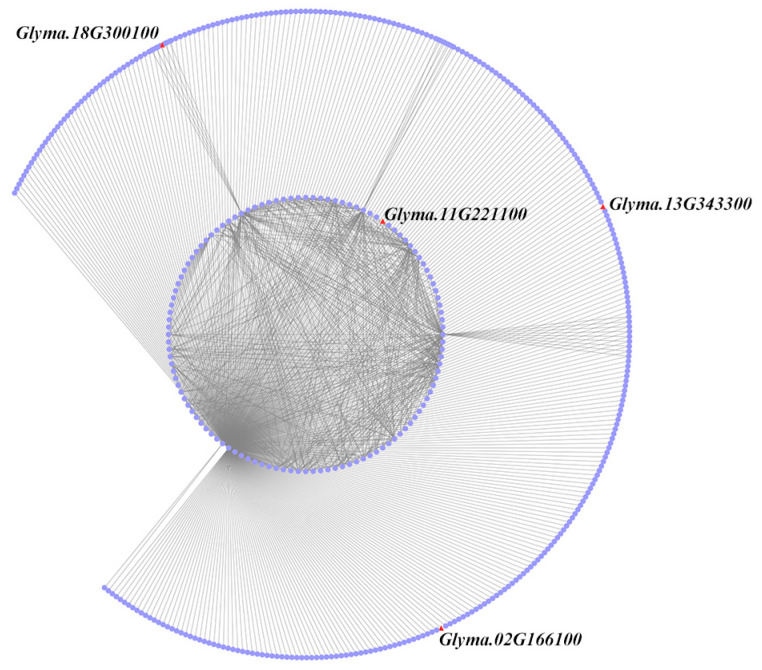
Co-expression network analysis of candidate genes in the MEblack module.

Furthermore, in order to obtain key genes that regulate oil synthesis, the genes in the MEblack module were screened based on a correlation greater than 0.57 as the threshold. A total of 863 linear networks were obtained and visualized using Cytoscape 3.9.1 software. As shown in Figure 5, four genes were identified in the GWAS results, including *Glyma.18G300100*, *Glyma.11G221100*, *Glyma.13G343300*, and *Glyma.02G166100*. Further analysis of candidate gene expression patterns revealed that the *Glyma.18G300100*, *Glyma.13G343300*, and *Glyma.02G166100* genes upregulated expression, and the *Glyma.11G221100* gene downregulated expression (Figure S4). Based on co-expression networks, the genes identified by the GWAS results are significantly correlated with other genes. *Glyma.18G070600* and *Glyma.18G300100* were found to be significantly correlated (r > 0.58). *Glyma.11G221100*, *Glyma.04G062400*, *Glyma.18G070600*, *Glyma.04G130300*, *Glyma.07G273900*, and *Glyma.09G212100* were found to be significantly correlated (r > 0.57). *Glyma.13G343300* and *Glyma.18G070600* were found to be significantly correlated (r > 0.57). *Glyma.02G166100* and *Glyma.18G070600* were found to be significantly correlated (r > 0.57) (Figure 5, Table S2).

### 2.6. Gene-Based Association and Haplotype Analysis of Candidate Genes

**Figure 6:**
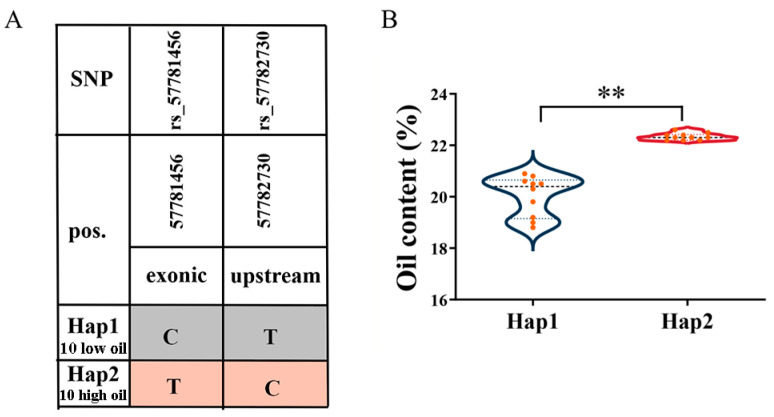
Haplotypes analysis of genes with variations related to oil content. (**A**) Statistical analysis of locus variations in two haplotypes of *Glyma*.*18G300100*. (**B**) Comparison of oil content between two different haplotypes in 20 soybean germplasms. ** indicates significance at *p* < 0.01.

To further elucidate the association between candidate genes and oil content, gene-based associations and haplotype analysis were calculated using the GLM method. The SNP extraction of the *Glyma.13G343300* gene identified six SNPs, and subsequent correlation analysis revealed no significant association between these SNPs and oil content (−log(*p)* < 2.5). Similarly, the SNPs extracted from *Glyma.11G221100* and *Glyma.02G166100* were found to be three and two SNPs, respectively, and there was no significant correlation with oil content (−log(*p*) < 2.5). However, based on the association analysis, two SNPs were identifed in the exonic and upstream regions of *Glyma.18G300100*, and the SNP variation distance between exonic and upstream regions was 1273 bp (Figure 6A). The variation in the CDS region occurs on the first exonic region and the variation in exonic region belongs to synonym mutation and has no effect on protein change. The SNP markers rs57781456 and rs57782730 showed a significant association with oil content (−log(*p*) > 2.5). *Glyma.18G300100* was divided into two predominant haplotypes in the studied accessions. The oil content of haplotype 1 is significantly lower than that of haplotype 2 (Figure 6B).

## 3. Discussion

Soybean is a significant economic crop and a primary source of vegetable oil [47]. Previous studies have found that many QTN loci are significantly associated with oil content. In this study, we performed genome sequencing on 227 soybean germplasm samples, and a total of 23,131 high-quality markers were identified. Of those, 44 SNPs were found to have significant association with oil content. A total of six, four, and 34 significant QTN loci were identified in Xiangyang, Hulan, and Acheng, respectively. In recent years, the combined analysis of GWAS and WGCNA has been utilized to identify some novel genes. For example, four candidate genes were identified through the integration of GWAS and WGCNA [48]. This study identified four candidate genes (*Glyma.18G300100*, *Glyma.11G221100*, *Glyma.13G343300*, and *Glyma.02G166100*) associated with oil content through the integration of the GWAS and WGCNA methods.

The GWAS is extensively employed for the analysis of the genetic basis of complex traits, and the identification of numerous candidate genes associated with the regulation of controlling target traits has been accomplished. Previous researchers have conducted GWAS analysis on unsaturated fatty acids (FA), utilizing a panel of 30,000 SNPs, and found nine, five, and five QTNs associated with the levels of linoleic acid (LLA), linolenic acid (LNA), and oleic acid (OA), respectively [49]. A GWAS was conducted to analyze the agronomic traits (plant height, number of nodes on main stem, branch number, stem diameter, and 100-seed weight) of 133 soybean germplasms, where a total of 59 SNPs were detected in at least two environments, and 15 candidate genes were further identified [50]. Previous studies conducted GWAS analysis on the 100-seed weight of 185 soybean varieties, where a total of 31 significant QTNs were identified, and further screening revealed 237 candidate genes related to 100-seed weight [51]. To evaluate the accuracy of SNPs in this study, the SNPs identified in this study were compared with previously published QTLs/SNPs. In this study, 44 QTNs related to oil content were identified in three environments in 2019. Meanwhile, the Acheng region exhibits the most significant loci, involving 34 loci. The higher number of significant QTL loci in the Acheng region compared to the other two regions may potentially be attributed to environmental factors. Out of these 44 QTNs, 26 QTNs were found to overlap with or be in close proximity to the previously identified oil content QTL. Two QTNs (rs13422 of Chr.13 and rs10231 of Chr.10) were significantly associated with oil content, and the association between locus rs13422 and oil content has been previously reported. Meanwhile, a total of 18 novel QTNs associated with oil content were identified.

Although GWAS analysis can strongly identify significant SNP–trait relationships, it may not accurately determine candidate genes. Therefore, the integrated GWAS and WGCNA joint analysis strategies can enhance the identification of candidate genes. Azam et al. (2023) used a combination of GWAS and WGCNA methodologies to identify four hub genes (*Glyma.11G108100*, *Glyma.11G107100*, *Glyma.11G106900*, and *Glyma.11G109100*) involved in TIF accumulation in soybean [52]. Li et al. (2021) used a combination of GWAS and WGCNA methodologies to identify eight candidate genes regulating root growth in rapeseed [53]. In this study, four candidate genes (*Glyma.18G300100*, *Glyma.11G221100*, *Glyma.13G343300*, and *Glyma.02G166100*) involved in oil synthesis were identified through the integrated GWAS and WGCNA analysis. The *Glyma.13G343300* gene encoded the E3 ubiquitin ligase protein, which is homologous to Arabidopsis *AT2G31510*, and was shown to be involved in promoting seedling oleosin degradation and lipid droplet mobilization [54]. The remaining three genes may be novel genes regulating oil synthesis. The *Glyma.11G221100* gene encoded phosphoribosylformylglycinamidine, according to reports that the gene can promote the expression of flower organs [55]. The *Glyma.02G166100* gene encoded an unknown protein. In addition, the present study provides SNP markers for the purpose of soybean oil synthesis breeding. In this study, the *Glyma.18G300100* gene was identified to possess two haplotypes in the exonic and upstream regions, including *Glyma.18G300100* Hap1 and Hap2. The oil content of haplotype 2 exhibits a significantly higher amount than that of haplotype 1, and the result shows that *Glyma.18G300100* genes beneficial to haplotypes might be valuable for molecular assistant selection (MAS) of the oil content of soybean. Meanwhile, the expression level of the *Glyma.18G300100* gene was found to be significantly higher in high oil soybean materials compared to low oil soybean materials. The reason for the variation of *Glyma.18G300100* expression between different materials remains to be further explored.

We conducted GWAS analysis on the oil content of 227 soybean seeds and further found that 44 significant SNPs loci were detected. Through the integrated GWAS and WGCNA analysis, we identified four potential candidate genes (*Glyma.18G300100*, *Glyma.11G221100*, *Glyma.13G343300*, and *Glyma.02G166100*). The haplotype analysis of candidate genes further revealed that *Glyma.18G300100* was divided into two haplotypes, and the oil content of haplotype 2 was significantly higher than that of haplotype 1. Therefore, variations in the exonic and upstream regions of *Glyma.18G300100* can help us provide a basis for MAS of soybean oil content.

## 4. Materials and Methods

### 4.1. Plant Materials

This study used 227 soybean germplasm resources as experimental materials (Table S3). All materials were planted in three locations in Harbin, including Xiangyang, Hulan, and Acheng (45.80° N, 126.53° E) in 2019. We used a single-row plot (3 m long, 0.65 m between rows, 34 plants per row) and repeated three times for each location. After the soybeans had fully matured, we randomly selected 10 mature soybean plants from each row at each location. We put the mature soybean seeds into the sample tank, and seed oil content was quantified using the Infratec 1241 NIR Grain Analyzer (FOSS, Hoganas, Sweden).

### 4.2. DNA Isolation and SNP Genotyping Data Collection

Genomic DNA was extracted from the soybean samples using the CTAB method, and the quality of extraction was assessed [56]. The isolated high-quality DNA was performed using specific-site amplification fragment sequencing (SLAF-seq) [57]. The restriction endonucleases MseI and HaeIII (Thermo Fisher Scientific Inc., Waltham, MA, USA) were selected to generate a minimum of 50,000 sequencing tags per tested sample, ranging in length from approximately 300 bp to 500 bp. The acquired tags were uniformly distributed across the distinct genomic regions of all 20 soybean chromosomes. The sequencing libraries of each tested samples were performed based on the sequencing tags. The Illumina Genome Analyzer II system (Illumina Inc., San Diego, CA, USA) was employed in conjunction with a barcode method to generate 45 bp sequence reads at both ends of the sequencing tags from each accession library. The Short Oligonucleotide Alignment Program 2 (SOAP2) software was employed for aligning the raw paired-end reads to the reference genome of soybean (Glycine max Wm82. a2. v1) [58]. The SAMtools48 (Version: 0.1.18) software was utilized for the conversion of mapping results into BAM format, facilitating the efficient filtration of unmapped and non-unique reads [59,60]. Quality control of genotype data was performed using PLINK 1.9 software (--maf 0.05 --geno 0.1) (http://pngu.mgh.harvard.edu/purcell/plink/) (accessed on 21 November 2023).

For twenty lines, the genome resequencing was performed on an Illumina HiSeq 2000 sequencer, generating paired-end reads with a depth of 10-fold. These reads were then aligned to the soybean Williams 82 reference genome (Glyma.Wm82. a2) using BWA [59]. The SAMtools48 software was utilized to convert the mapping results into the BAM format and perform filtration of unmapped and non-unique reads [60]. The Picard package (https://sourceforge.net/p/picard/wiki/Main_Page/) (accessed on 21 November 2023) was utilized to eliminate duplicated reads. The BEDtools coverageBed program was utilized to calculate the sequence alignment coverage [61]. A sequence was considered absent if the coverage was below 90%, and present if it exceeded 90%. SNP detection was carried out using the Genome Analysis Toolkit and SAMtools [60,62]. The SNP annotation was conducted based on the soybean genome through the ANNOVAR package [63].

### 4.3. Population Structure Evaluation, Linkage Disequilibrium (LD) and Genome-Wide Association Study (GWAS)

The soybean oil content association signals were found based on 23,150 SNPs from 227 soybean germplasm resources with the compressed mixed linear model (CMLM) model of the R (version 4.2.3) software GAPIT package [64], using the first three PCA (principal components analysis) as covariates for association analysis to reduce false positives. Significant SNP markers were selected based on the threshold horizontal line with −log (*p*) > 4 as a significant correlation. A total of 23,150 high-quality SNPs (MAF > 0.05, missing data < 10%) were selected. The Manhattan plots depicting the oil contents for each of the three environments were established using GAPIT [64]. An LDdecay diagram was established through PopLDdecay [65]. The R^2^ value of LD was calculated using Tassel software [66]. The decay point is determined by taking half of the maximum LD value. The SoyBase database (http://www.soybase.org/) (accessed on 23 November 2023) was utilized for the prediction and annotation of candidate genes. The GO (http://www.geneontology.org/) (accessed on 23 November 2023) enrichment analysis was conducted based on the SoyBase database. The KEGG (https://www.kegg.jp/) (accessed on 23 November 2023) database was utilized for conducting pathway enrichment analysis of candidate genes.

### 4.4. Weighted Gene Co-Expression Network Analysis (WGCNA)

WGCNA analysis was conducted using transcriptome data from 30 soybean varieties (including 15 extremely high oil and 15 extremely low oil soybean varieties), which were obtained from Zhao et al. (2023) [67]. Total RNA was extracted from the R6 stage of soybean development using TRIzol reagent (Invitrogen). The purity and concentration of RNA samples were assessed, followed by the construction of the library. The construction of the cDNA library was performed utilizing the Illumina HiSeq sequencing platform. The high-quality readings were aligned to the reference genomes (Glycine max Wm82.a2.v1) using the Hisat2 v2.0.5 software. Using the R software WGCNA package to construct a weighted gene co-expression network, the gene expression profile matrix was derived from the gene expression levels of all samples [68]. Firstly, clustering analysis was performed subsequent to the exclusion of samples exhibiting low correlation or those that were unable to be grouped on the dendrogram. The pick Soft Threshold function within the WGCNA package was utilized to calculate the Soft Threshold, ensuring compliance with the prerequisite of scale-free network distribution. The value of the Threshold parameter β was selected at the point where the fitting curve first approached 0.9. Subsequently, the correlation-based association between phenotype and gene modules was performed to generate an adjacency matrix based on the β value. Further transforming the adjacency matrix into a topological overlap matrix (TOM), a gene connectivity network was constructed. Finally, the gene modules were generated and clustered using the dynamic tree cut method, which is based on the eigengenes (ME) of each module. The co-expression networks were visualized using the Cytoscape 3.10.1 package.

### 4.5. Prediction of Candidate Genes Controlling Oil Content

The genes in the upstream and downstream 100 kb genomic regions of each significant SNP were selected as candidate genes. The identified variations in the exonic, 5′ UTR, and 3′ UTR regions of 10 high oil content soybean materials’ and 10 low oil content soybean materials’ candidate genes from genomic resequencing data were obtained. The gene-based association analysis was performed using the General Linear Model (GLM) method to determine SNPs or haplotypes associated with oil content by TASSEL 5.0 software [66]. SNPs with threshold −log10 (*p*) ≥ 2.5 were set as having a significant association.

### 4.6. Quantitative Real-Time PCR

The expression levels of candidate genes were analyzed by quantitative real-time PCR. Total RNA extracted from soybean developmental seeds was obtained using TRIzol reagent (Invitrogen), and further generation of cDNA was performed through ReverTra Ace qPCR RT Master Mix (TOYOBO, Osaka, Japan). The ABI 7500 fast real-time PCR platform was applied for SYBR Select Master Mix RT-PCR (TOYOBO, Osaka, Japan). Relative expression levels were calculated by the 2^−ΔΔCT^ method. The *GmACTIN4* gene was applied as the internal control. All qRT-PCR primers are collected in Table S4.

## Figures and Tables

**Table 1:** Statistical analysis of oil content in soybean.

Trait	Location	Min (%)	Max (%)	Mean (%)	SD	CV (%)
Oil content	Xiangyang	16.9	22.6	21.07	0.88	4.18%
	Hulan	17	23.8	21.28	0.77	3.62%
	Acheng	17	24.6	21.17	0.99	4.68%

**Table 2:** Single nucleotide polymorphisms (SNPs) associated with oil content of soybean and known QTLs overlapping with peak SNP.

Locus Name	Env	Chr	Position	Effect	−Log10 (*p*)	Known QTLs
rs334	E1	Chr01	13162732	0.6	4.17	[30]
rs1773	E3	Chr02	24938426	1.31	5.01	[31]
rs1864	E3	Chr02	30664940	1.31	4.74	
rs2905	E2	Chr03	26858965	0.61	4.33	[32]
rs2705	E3	Chr03	16624895	−1.43	5.16	
rs3413	E3	Chr03	45485942	−0.88	4.03	
rs5244	E1	Chr05	29415473	0.53	4.09	
rs4932	E3	Chr05	6626018	−1.28	4.75	
rs6151	E3	Chr06	20915980	−1.46	4.71	
rs6995	E2	Chr07	3914634	−0.44	4.15	[31]
rs7023	E3	Chr07	5265620	−1.34	4.35	[31]
rs7610	E3	Chr07	31259735	1.11	4.24	[33]
rs8311	E3	Chr08	16533608	−1.6	5.31	[34]
rs8312	E3	Chr08	16533609	−1.39	4.63	[34]
rs8750	E3	Chr08	39896867	1.07	4.14	
rs8346	E3	Chr08	17827024	1.17	4.11	[35]
rs9475	E1	Chr09	18054299	−0.44	4.06	
rs9263	E3	Chr09	8713565	1.44	5.61	[31,36]
rs9049	E3	Chr09	2815266	−1.1	4.2	[31]
rs9227	E3	Chr09	7692237	−1.1	4.14	[31]
rs10231	E3	Chr10	120009	−2.04	6.94	
rs10297	E3	Chr10	2562781	1.34	4.45	
rs11438	E3	Chr11	2041489	−1.39	5.36	
rs11998	E3	Chr11	31702175	0.99	4.17	
rs13933	E2	Chr13	43375152	0.35	4.09	
rs13422	E3	Chr13	24269755	−2.19	7.32	[37]
rs13907	E3	Chr13	42167470	1.6	5.35	[38]
rs13704	E3	Chr13	33703570	−1.25	4.58	[39]
rs14506	E3	Chr14	21694109	1.43	5.03	[40]
rs14799	E3	Chr14	40317213	−1.7	4.83	
rs14524	E3	Chr14	22596638	1.0	4.16	[41]
rs15289	E3	Chr15	10698617	−1.65	5.17	[38]
rs16850	E1	Chr16	3534128	0.57	4.28	[42]
rs17991	E1	Chr17	3109140	0.83	4.86	
rs18353	E3	Chr17	16440855	−1.84	6.06	
rs18145	E3	Chr17	9725100	−0.74	4.39	[31]
rs17989	E3	Chr17	3023762	1.18	4.06	
rs20739	E1, E2	Chr18	57800686	−1.14	6.46	
rs20078	E2	Chr18	36425921	0.53	4.12	[40]
rs19314	E3	Chr18	9916890	−2.07	6.19	[38]
rs20112	E3	Chr18	38930008	1.36	5.64	[40]
rs20110	E3	Chr18	38876843	1.0	4.09	[40]
rs20814	E3	Chr19	2256179	1.02	4.07	[39]
rs22223	E3	Chr20	4151851	−1.45	4.44	[33]

## Data Availability

Data are contained within the article and Supplementary Materials.

## Supplementary Materials

The following supporting information can be downloaded at: https://www.mdpi.com/article/10.3390/plants13101351/s1, Figure S1: Soybean resources and genotype characteristics. (A) The density distribution of SNPs. (B) The first three principal components reflected by 23,150 SNPs used in the GWAS. (C) Population structure of soybean germplasm. (D) A heatmap of the kinship matrix of the 267 soybean accessions. Figure S2: LD decay of the GWAS population. Figure S3: KEGG enrichment of candidate genes. Figure S4: Analysis of candidate genes by qRT-PCR. Table S1: Genes in 100 kbp flanking regions of peak SNP associated with oil content of soybean. Table S2: Co-expression network correlation coefficient. Table S3: The information of soybean association panel. Table S4: Primers used for qRT-PCR.

## References

[1] Hooker J.C., Smith M., Zapata G., Charette M., Luckert D., Mohr R.M., Daba K.A., Warkentin T.D., Hadinezhad M., Barlow B. (2023). Differential gene expression provides leads to environmentally regulated soybean seed protein content. Front Plant Sci..

[2] Li H., Peng Z., Yang X., Wang W., Fu J., Wang J., Han Y., Chai Y., Guo T., Yang N. (2013). Genome-wide association study dissects the genetic architecture of oil biosynthesis in maize kernels. Nat. Genet..

[3] Miao L., Yang S., Zhang K., He J., Wu C., Ren Y., Gai J., Li Y. (2020). Natural variation and selection in GmSWEET39 affect soybean seed oil content. New Phytol..

[4] Clemente T.E., Cahoon E.B. (2009). Soybean oil: Genetic approaches for modification of functionality and total content. Plant Physiol..

[5] Lee J.D., Bilyeu K.D., Pantalone V.R., Gillen A.M., So Y.S., Shannon J.G. (2012). Environmental stability of oleic acid concentration in seed oil for soybean lines with FAD2-1A and FAD2-1B mutant genes. Crop. Sci..

[6] Gibellini F., Smith T.K. (2010). The Kennedy pathway—De novo synthesis of phosphatidylethanolamine and phosphatidylcholine. IUBMB Life..

[7] Cao J., Li J., Li D., Tobin J.F., Gimeno R.E. (2006). Molecular identification of microsomal acyl-CoA: Glycerol-3-phosphate acyltransferase, a key enzyme in *de novo* triacylglycerol synthesis. Proc. Natl. Acad. Sci. USA.

[8] Zhang D., Zhang H., Hu Z., Chu S., Yu K., Lv L., Yang Y., Zhang X., Chen X., Kan G. (2019). Artificial selection on GmOLEO1 contributes to the increase in seed oil during soybean domestication. PLoS Genet..

[9] Liu J., Hao W., Liu J., Fan S., Zhao W., Deng L., Wang X., Hu Z., Hua W., Wang H. (2019). A novel chimeric mitochondrial gene confers cytoplasmic effects on seed oil content in polyploid rapeseed (*Brassica napus*). Mol. Plant..

[10] Baud S., Wuillème S., To A., Rochat C., Lepiniec L. (2009). Role of WRINKLED1 in the transcriptional regulation of glycolytic and fatty acid biosynthetic genes in Arabidopsis. Plant J..

[11] Baud S., Mendoza M.S., To A., Harscoët E., Lepiniec L., Dubreucq B. (2007). WRINKLED1 specifies the regulatory action of LEAFY COTYLEDON2 towards fatty acid metabolism during seed maturation in Arabidopsis. Plant J..

[12] To A., Joubès J., Barthole G., Lécureuil A., Scagnelli A., Jasinski S., Lepiniec L., Baud S. (2012). WRINKLED transcription factors orchestrate tissue-specific regulation of fatty acid biosynthesis in Arabidopsis. Plant Cell..

[13] Pelletier J.M., Kwong R.W., Park S., Le B.H., Baden R., Cagliari A., Hashimoto M., Munoz M.D., Fischer R.L., Goldberg R.B. (2017). LEC1 sequentially regulates the transcription of genes involved in diverse developmental processes during seed development. Proc. Natl. Acad. Sci. USA..

[14] Manan S., Ahmad M.Z., Zhang G., Chen B., Haq B.U., Yang J., Zhao J. (2017). Soybean LEC2 regulates subsets of genes involved in controlling the biosynthesis and catabolism of seed storage substances and seed development. Front. Plant Sci..

[15] Lee H.G., Kim H., Suh M.C., Kim H.U., Seo P.J. (2018). The MYB96 transcription factor regulates triacylglycerol accumulation by activating DGAT1 and PDAT1 expression in Arabidopsis seeds. Plant Cell Physiol..

[16] Song Q., Li Q., Liu Y., Zhang F., Ma B., Zhang W., Man W., Du W., Wang G., Chen S. (2013). Soybean GmbZIP123 gene enhances lipid content in the seeds of transgenic Arabidopsis plants. J. Exp. Bot..

[17] Zhou H., Xia D., Li P., Ao Y., Xu X., Wan S., Li Y., Wu B., Shi H., Wang K. (2021). Genetic architecture and key genes controlling the diversity of oil composition in rice grains. Mol. Plant..

[18] Jin H., Yang X., Zhao H., Song X., Tsvetkov Y.D., Wu Y., Gao Q., Zhang R., Zhang J. (2023). Genetic analysis of protein content and oil content in soybean by genome-wide association study. Front. Plant Sci..

[19] Goettel W., Zhang H., Li Y., Qiao Z., Jiang H., Hou D., Song Q., Pantalone V.R., Song B.H., Yu D. (2022). POWR1 is a domestication gene pleiotropically regulating seed quality and yield in soybean. Nat. Commun..

[20] Duan Z., Zhang M., Zhang Z., Liang S., Fan L., Yang X., Yuan Y., Pan Y., Zhou G., Liu S. (2022). Natural allelic variation of GmST05 controlling seed size and quality in soybean. Plant Biotechnol. J..

[21] Qi Z., Guo C., Li H., Qiu H., Li H., Jong C., Yu G., Zhang Y., Hu L., Wu X. (2024). Natural variation in Fatty Acid 9 is a determinant of fatty acid and protein content. Plant Biotechnol. J..

[22] Li B., Peng J., Wu Y., Hu Q., Huang W., Yuan Z., Tang X., Cao D., Xue Y., Luan X. (2023). Identification of an important QTL for seed oil content in soybean. Mol. Breed..

[23] Yu J., Zhu C., Xuan W., An H., Tian Y., Wang B., Chi W., Chen G., Ge Y., Li J. (2023). Genome-wide association studies identify OsWRKY53 as a key regulator of salt tolerance in rice. Nat Commun..

[24] Liang Q., Chen L., Yang X., Yang H., Liu S., Kou K., Fan L., Zhang Z., Duan Z., Yuan Y. (2022). Natural variation of Dt2 determines branching in soybean. Nat. Commun..

[25] Hwang E.Y., Song Q., Jia G., Specht J.E., Hyten D.L., Costa J., Cregan P.B. (2014). A genome-wide association study of seed protein and oil content in soybean. BMC Genomics..

[26] Cao Y., Li S., Wang Z., Chang F., Kong J., Gai J., Zhao T. (2017). Identification of major quantitative trait loci for seed oil content in soybeans by combining linkage and genome-wide association mapping. Front. Plant Sci..

[27] Zeng A., Chen P., Korth K., Hancock F., Pereira A., Brye K., Wu C., Shi A. (2017). Genome-wide association study (GWAS) of salt tolerance in worldwide soybean germplasm lines. Mol. Breed..

[28] Chen Q., Zhang R., Li D., Wang F. (2021). Transcriptomic and coexpression network analyses revealed pine chalconesynthase genes associated with pine wood nematode infection. Int. J. Mol. Sci..

[29] Yang J., Ren Y., Zhang D., Chen X., Huang J., Xu Y., Aucapiña C.B., Zhang Y., Miao Y. (2021). Transcriptome-Based WGCNA analysis reveals regulated metabolite fluxes between floral color and scent in narcissus tazetta flower. Int. J. Mol. Sci..

[30] Hyten D.L., Pantalone V.R., Sams C.E., Saxton A.M., Landau-Ellis D., Stefaniak T.R., Schmidt M.E. (2004). Seed quality QTL in a prominent soybean population. Theor. Appl. Genet..

[31] Mao T., Jiang Z., Han Y., Teng W., Zhao X., Li W., Morris B. (2013). Identification of quantitative trait loci underlying seed protein and oil contents of soybean across multi-genetic backgrounds and environments. Plant Breed..

[32] Wang X., Jiang G., Green M., Scott R.A., Song Q., Hyten D.L., Cregan P.B. (2014). Identification and validation of quantitative trait loci for seed yield, oil and protein contents in two recombinant inbred line populations of soybean. Mol. Genet. Genomics..

[33] Teuku T., Satoshi W., Naoki Y., Kyuya H. (2003). Analysis of Quantitative Trait Loci for Protein and Lipid Contents in Soybean Seeds Using Recombinant Inbred Lines. Breed. Sci..

[34] Panthee D.R., Pantalone V.R., Saxton A.M. (2006). Modifier QTL for fatty acid composition in soybean oil. Euphytica.

[35] Li H., Zhao T., Wang Y., Yu D., Chen S., Zhou R., Gai J. (2011). Genetic structure composed of additive QTL, epistatic QTL pairs and collective unmapped minor QTL conferring oil content and fatty acid components of soybeans. Euphytica.

[36] Mansur L.M., Lark K.G., Kross H., Oliveira A. (1993). Interval mapping of quantitative trait loci for reproductive, morphological, and seed traits of soybean (*Glycine max* L.). Theor. Appl. Genet.

[37] Bachlava E., Dewey R.E., Burton J.W., Cardinal A.J. (2009). Mapping and comparison of quantitative trait loci for oleic acid seed content in two segregating soybean populations. Crop. Sci..

[38] Reinprecht Y., Poysa V.W., Yu K., Rajcan I., Ablett G.R., Pauls K.P. (2006). Seed and agronomic QTL in low linolenic acid, lipoxygenase-free soybean (*Glycine max* (L.) Merrill) germplasm. Genome..

[39] Eskandari M., Cober E.R., Rajcan I. (2013). Genetic control of soybean seed oil: I. QTL and genes associated with seed oil concentration in RIL populations derived from crossing moderately high-oil parents. Theor. Appl. Genet..

[40] Qi Z., Wu Q., Han X., Sun Y., Du Y., Liu C., Jiang H., Hu G., Chen Q. (2011). Soybean oil content QTL mapping and integrating with meta-analysis method for mining genes. Euphytica.

[41] Han Y., Wang W., Zhao Y., Wu X., Li L., Li D., Li W. (2015). Unconditional and conditional QTL underlying the genetic interrelationships between soybean seed isoflavone, and protein or oil contents. Plant Breed..

[42] Kabelka E.A., Diers B.W., Fehr W.R., Leroy A.R., Baianu I.C., You T., Neece D.J., Nelson R.L. (2004). Putative alleles for increased yield from soybean plant introductions. Crop. Sci..

[43] Zan X., Cui F., Sun J., Zhou S., Song Y. (2019). Novel dual-functional enzyme Lip10 catalyzes lipase and acyltransferase activities in the oleaginous fungus mucor circinelloides. J. Agric. Food Chem..

[44] Ding L., Guo X., Li M., Fu Z., Yan S., Zhu K., Wang Z., Tan X. (2019). Improving seed germination and oil contents by regulating the GDSL transcriptional level in *Brassica napus*. Plant Cell Rep..

[45] Stenback K.E., Flyckt K.S., Hoang T., Campbell A.A., Nikolau B.J. (2022). Modifying the yeast very long chain fatty acid biosynthetic machinery by the expression of plant 3-ketoacyl CoA synthase isozymes. Sci. Rep..

[46] Zhao Y., Huang Y., Wang Y., Cui Y., Liu Z., Hua J. (2018). RNA interference of GhPEPC2 enhanced seed oil accumulation and salt tolerance in Upland cotton. Plant Sci..

[47] Liu X., Jin J., Wang G., Herbert S.J. (2008). Soybean yield physiology and development of high-yielding practices in northeast China. Field Crop. Res..

[48] Liang T., Qing C., Liu P., Zou C., Yuan G., Pan G., Shen Y., Ma L. (2022). Joint GWAS and WGCNA uncover the genetic control of calcium accumulation under salt treatment in maize seedlings. Physiol. Plant..

[49] Leamy L.J., Zhang H., Li C., Chen C., Song B. (2017). A genome-wide association study of seed composition traits in wild soybean (*Glycine soja*). BMC Genomics..

[50] Zhang X., Ding W., Xue D., Li X., Zhou Y., Shen J., Feng J., Guo N., Qiu L., Xing H. (2021). Genome-wide association studies of plant architecture-related traits and 100-seed weight in soybean landraces. BMC Genomics..

[51] Zhao X., Dong H., Chang H., Zhao J., Teng W., Qiu L., Li W., Han Y. (2019). Genome wide association mapping and candidate gene analysis for hundred seed weight in soybean [*Glycine max* (L.) Merrill]. BMC Genomics..

[52] Azam M., Zhang S., Li J., Ahsan M., Agyenim-Boateng K.G., Qi J., Feng Y., Liu Y., Li B., Qiu L. (2023). Identification of hub genes regulating isoflavone accumulation in soybean seeds via GWAS and WGCNA approaches. Front. Plant Sci..

[53] Li K., Wang J., Kuang L., Tian Z., Wang X., Dun X., Tu J., Wang H. (2021). Genome-wide association study and transcriptome analysis reveal key genes affecting root growth dynamics in rapeseed. Biotechnol. Biofuels..

[54] Wu P., Gao H., Liu J., Kosma D.K., Lü S., Zhao H. (2021). Insight into the roles of the ER-associated degradation E3 ubiquitin ligase HRD1 in plant cuticular lipid biosynthesis. Plant Physiol. Biochem..

[55] Vaghchhipawala Z.E., Schlueter J.A., Shoemaker R.C., Mackenzie S.A. (2004). Soybean FGAM synthase promoters direct ectopic nematode feeding site activity. Genome..

[56] Han Y., Zhao X., Cao G., Wang Y., Li Y., Liu D., Qiu L., Zheng H., Li W. (2015). Genetic characteristics of soybean resistance to HG type 0 and HG type 1.2.3.5.7 of the cyst nematode analyzed by genome-wide association mapping. BMC Genomics..

[57] Sun X., Liu D., Zhang X., Li W., Liu H., Hong W., Jiang C., Guan N., Ma C., Zeng H. (2013). SLAF-seq: An efficient method of large-scale de novo SNP discovery and genotyping using high-throughput sequencing. PLoS ONE.

[58] Li R., Yu C., Li Y., Lam T.W., Yiu S.M., Kristiansen K., Wang J. (2009). SOAP2: An improved ultrafast tool for short read alignment. Bioinformatics.

[59] Li H., Durbin R. (2009). Fast and accurate short read alignment with Burrows-Wheeler transform. Bioinformatics.

[60] Li H., Handsaker B., Wysoker A., Fennell T., Ruan J., Homer N., Marth G., Abecasis G., Durbin R. (2009). The Sequence Alignment/Map format and SAMtools. Bioinformatics.

[61] Quinlan A.R. (2014). BEDTools: The Swiss-Army tool for genome feature analysis. Curr. Protoc. Bioinform..

[62] McKenna A., Hanna M., Banks E., Sivachenko A., Cibulskis K., Kernytsky A., Garimella K., Altshuler D., Gabriel S., Daly M. (2010). The Genome Analysis Toolkit: A MapReduce framework for analyzing next-generation DNA sequencing data. Genome Res..

[63] Wang K., Li M., Hakonarson H. (2010). ANNOVAR: Functional annotation of genetic variants from high-throughput sequencing data. Nucleic Acids Res..

[64] Lipka A.E., Tian F., Wang Q., Peiffer J., Li M., Bradbury P.J., Gore M.A., Buckler E.S., Zhang Z. (2012). GAPIT: Genome association and prediction integrated tool. Bioinformatics.

[65] Zhang C., Dong S., Xu J., He W., Yang T. (2019). PopLDdecay: A fast and effective tool for linkage disequilibrium decay analysis based on variant call format files. Bioinformatics.

[66] Bradbury P.J., Zhang Z., Kroon D.E., Casstevens T.M., Ramdoss Y., Buckler E.S. (2007). TASSEL: Software for association mapping of complex traits in diverse samples. Bioinformatics.

[67] Zhao X., Wang J., Xia N., Liu Y., Qu Y., Ming M., Zhan Y., Han Y., Zhao X., Li Y. (2023). Combined analysis of the metabolome and transcriptome provides insight into seed oil accumulation in soybean. Biotechnol. Biofuels Bioprod..

[68] Langfelder P., Horvath S. (2008). WGCNA: An r package for weighted correlation network analysis. BMC Bioinform..

