# Supplementary figures and images for: GWAS and WGCNA Analysis Uncover Candidate Genes Associated with Oil Content in Soybean
Source: Plants (Basel). 2024 May 14;13(10):1351. doi: 10.3390/plants13101351 (PMC11125034; doi:10.3390/plants13101351)

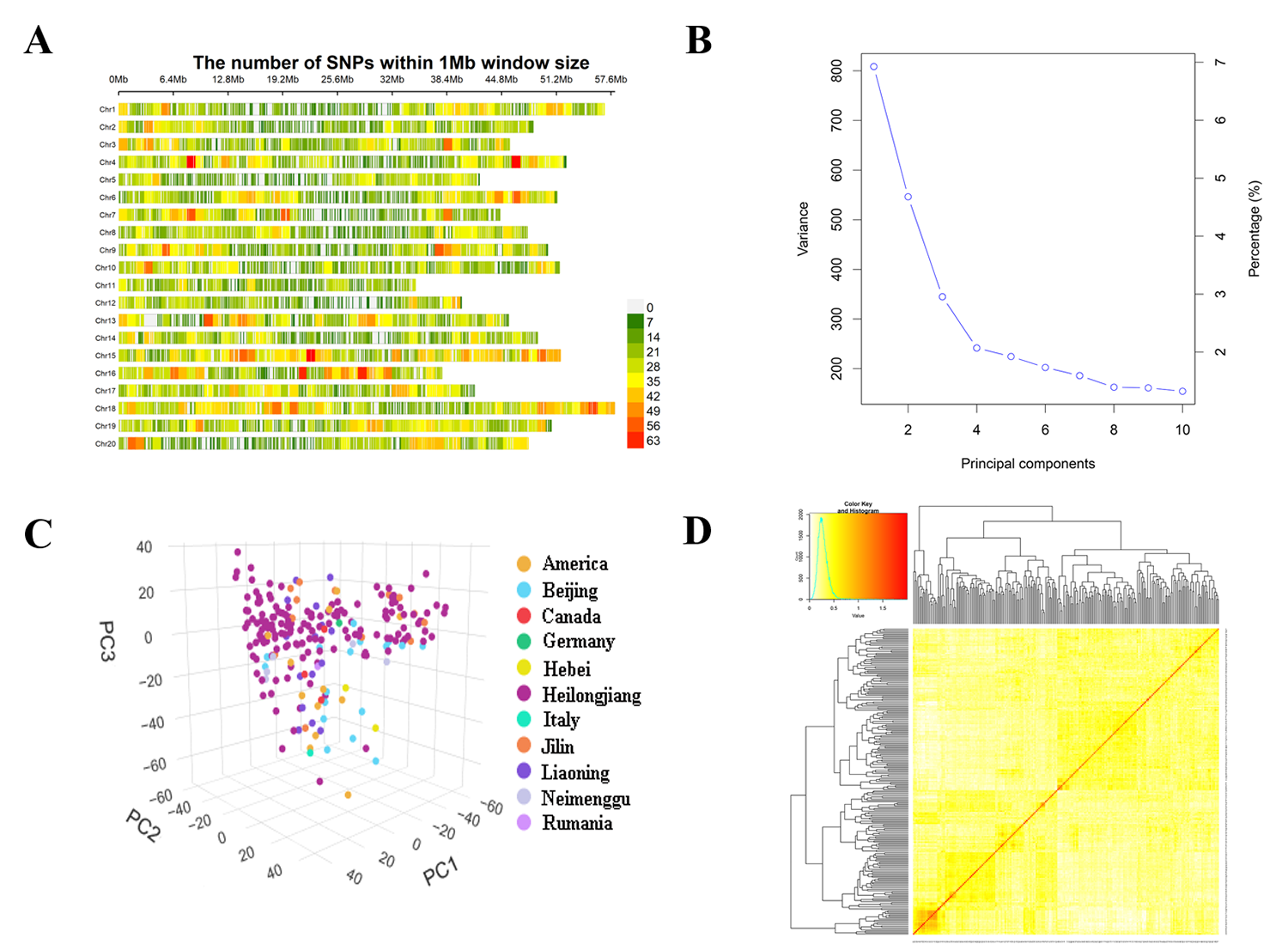

Supplement: Supplementary file 1 [file plants-13-01351-s001.zip › FigureS1.tif]

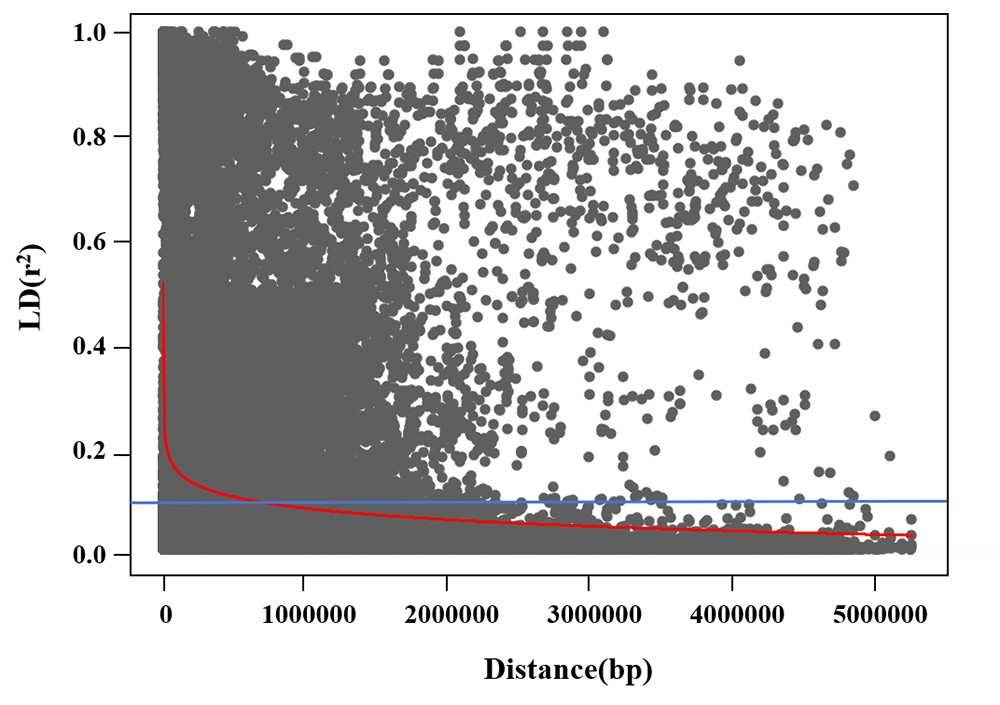

Supplement: Supplementary file 1 [file plants-13-01351-s001.zip › FigureS2.tif]

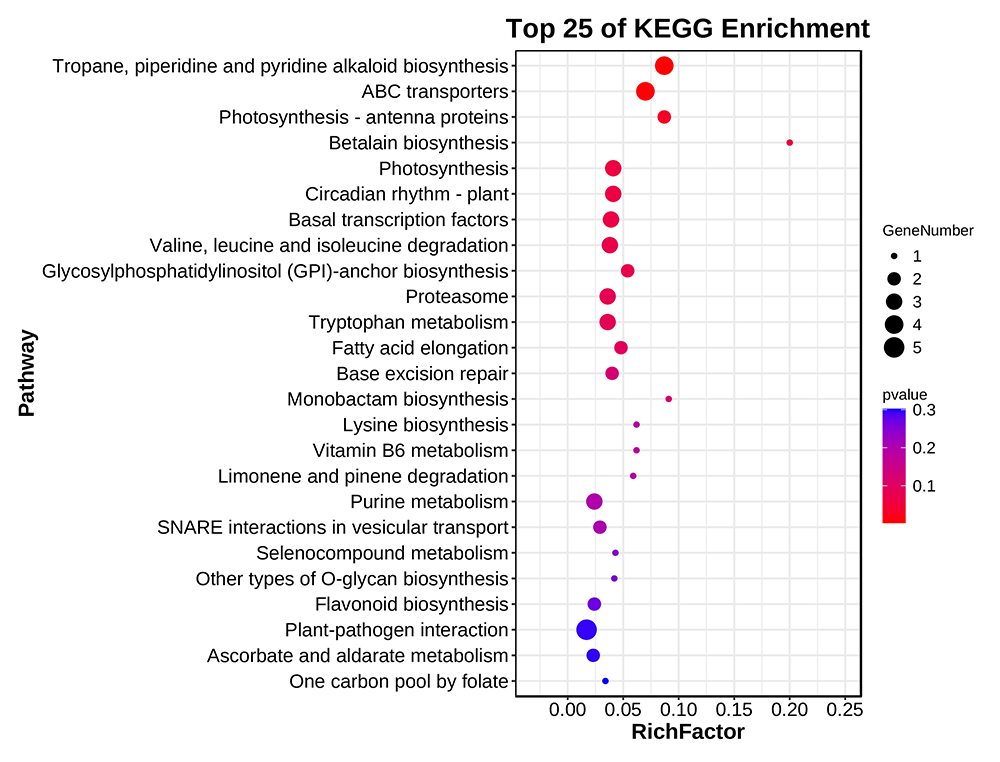

Supplement: Supplementary file 1 [file plants-13-01351-s001.zip › FigureS3.tif]

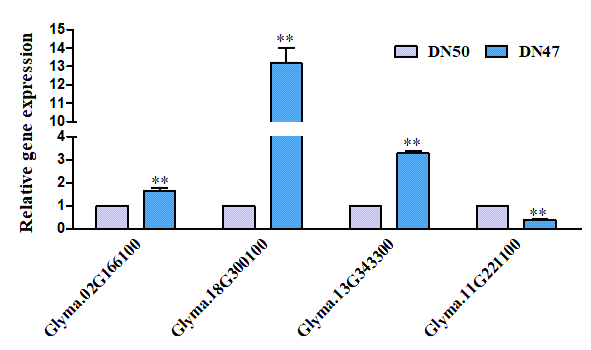

Supplement: Supplementary file 1 [file plants-13-01351-s001.zip › FigureS4.tif]
